# A GAITS-driven gap-to-deliverable accelerator for early-stage neurotechnology: design and early outcomes of the blueprint MedTech seedling program

**DOI:** 10.3389/fmedt.2026.1925746

**Published:** 2026-10-22

**Authors:** Evaristus A. Nwulia, Hasan Ali, Maria Mañanita S. Hipolito, Narayan Rai, Santosh Venkatesha, John Collins, Penny Ford Carleton

**Affiliations:** 1 Department of Psychiatry and Behavioral Sciences, Howard University College of Medicine, Washington, DC, United States; 2 Department of Electrical Engineering and Computer Science, Howard University College of Engineering and Architecture, Washington, DC, United States; 3 Whiting School of Engineering, Johns Hopkins University, Baltimore, MD, United States; 4 Center for Integration of Medicine and Innovative Technology, Massachusetts General Hospital, Boston, MA, United States

**Keywords:** blueprint MedTech, gaits, gap-to-deliverable, HealthTech innovation, medical device development, neurotechnology translation, NIH, translational readiness

## Abstract

**Background:**

Early-stage neurotechnology teams face a persistent structural barrier between scientific proof-of-concept and clinical/commercial viability. Traditional funding mechanisms, including investigator-initiated grants and early-stage SBIR/STTR awards, prioritize scientific novelty but may not address regulatory, clinical, market, and business challenges required for translation. Programs such as I-Corps and C3i improve commercialization literacy but primarily function as educational interventions. The NIH Blueprint MedTech (BPMT) Seedling Program addresses this gap by converting reviewer-identified commercialization weaknesses into structured, time-bounded work packages aligned with the Guidance and Impact Tracking System (GAITS), a commercialization maturity framework, and supported by experienced mentors.

**Objective:**

To describe the GAITS-driven Gap-to-Deliverable (G2G) acceleration model and characterize early program outcomes among neurotechnology teams participating in the BPMT Seedling Program.

**Methods:**

The six-month, mentor-centered Seedling Program operates within two NIH-funded neurotechnology incubator hubs: the Center for Innovative NeuroTech Advancement (CINTA) and NeuroTech Harbor (*N*TH). Each selected team receives $50,000 to address reviewer-identified commercialization gaps. Following multidisciplinary review, weaknesses are mapped to GAITS maturity indicators across Technology, Regulatory, Market/Business, and Clinical domains and converted into evidence-generating Work Packages. This descriptive program evaluation analyzed gap distributions across three cohorts (*N* = 58) and Deep Dive progression outcomes for two completed cohorts (*N* = 39).

**Results:**

GAITS-based review identified frequent commercialization gaps at program entry. Across all 58 teams, gaps were identified in team composition for 48/58 (82.8%), commercialization strategy for 47/58 (81.0%), work planning for 38/58 (65.5%), and regulatory strategy for 33/58 (56.9%). Among the two completed cohorts, 13/17 teams (76.5%; exact 95% CI, 50.1%–93.2%) in Cohort 1 and 18/22 teams (81.8%; 95% CI, 59.7%-94.8%) in Cohort 2 advanced to Blueprint MedTech Deep Dive evaluation; the combined progression rate was 31/39 (79.5%; 95% CI, 63.5%–90.7%). Cohort-specific rates did not differ statistically (Fisher exact *p* = 0.709).

**Conclusion:**

Among pre-screened teams with scientific or technical merit and addressable commercialization gaps, most advanced to internal Deep Dive evaluation after completing GAITS-aligned Work Packages. However, this uncontrolled evaluation of a selected cohort and program-specific endpoint does not establish causality or improved translational readiness. Prospective studies should incorporate standardized pre/post maturity measures, external downstream outcomes, and appropriate comparison groups to evaluate program effectiveness.

## 1 Introduction

The translation of early-stage neurotechnology from scientific proof-of-concept to clinically deployable, commercially viable products represent one of the most demanding challenges in biomedical innovation. Unlike advances in pharmaceuticals or basic research tools, medical devices and neurotechnologies must simultaneously satisfy a complex, interdependent matrix of clinical, regulatory, reimbursement, technical, and stakeholder requirements before they can reach patients (1, 2). This requirement is structural and non-negotiable: a device cannot proceed to first-in-human study without a defined regulatory pathway, a compelling clinical use case, validated user requirements, and a credible value proposition for payers and healthcare systems, regardless of its technical sophistication. Each of these domains demands specialized knowledge, iterative evidence generation, and strategic decision-making that extend well beyond the scientific training of most innovators (3, 4).

The consequences of this mismatch are well documented. Despite substantial federal investment in basic neuroscience and biomedical engineering, the vast majority of early-stage device concepts fail to advance beyond prototyping, not because of fundamental scientific deficiencies, but because the teams behind them lack structured access to commercialization guidance, domain expertise, and the regulatory and business fluency required to de-risk their technologies for downstream investment (5, 6). This failure mode is most acute at the pre-seed stage, where teams transition from academic discovery environments to the early demands of translational development. At this inflection point, the gap between scientific feasibility and commercial viability is widest, the evidence base for investment decisions is thinnest, and the cost of strategic missteps, choosing the wrong regulatory predicate, misidentifying the clinical use case, developing prototypes misaligned with real-world workflows, is highest. The resulting attrition has been widely described as the “valley of death” in biomedical translation (7).

Neurotechnology occupies a particularly challenging position within this landscape. Unlike many device categories, neurotechnologies must navigate the full spectrum of medical device regulatory complexity—spanning implantable systems, neuromodulation platforms, diagnostic algorithms, and rehabilitation devices, each with distinct FDA classification pathways, human factors requirements, and clinical validation demands (8, 9). Clinical adoption pathways are equally demanding: neurotechnologies typically require buy-in from multiple specialist communities, including neurologists, neurosurgeons, psychiatrists, and rehabilitation specialists, whose workflow, risk tolerance, and institutional constraints differ substantially (10). Reimbursement strategies must be established before clinical trials, as coverage uncertainty can collapse commercial viability even after successful regulatory clearance. These compounding uncertainties mean that early-stage neurotechnology teams who lack multi-domain commercialization infrastructure are structurally disadvantaged relative to the challenges ahead.

In response to these systemic bottlenecks, HealthTech Innovation Commercialization Programs emerged over the past two decades to provide structured, non-technical support to early-stage innovators. The National Science Foundation's Innovation Corps (I-Corps) program, introduced in 2011 and subsequently adapted for NIH-funded biomedical investigators, introduced customer discovery and Lean LaunchPad principles to academic device development, emphasizing iterative business model testing and value proposition refinement (11). In parallel, NIH's Concept to Clinic: Commercializing Innovation (C3i) program offered structured training in regulatory science, reimbursement strategy, and clinical development planning for medical device investigators transitioning toward the clinic (12). These programs made important contributions to commercialization literacy in academic settings and have been widely adopted across the biomedical research enterprise. However, they share a critical structural limitation: both are fundamentally *educational* interventions. They build conceptual knowledge and entrepreneurial skills but do not provide structured, standardized maturity models that assess multi-domain commercialization readiness, nor do they require teams to generate the regulatory, clinical, and market *evidence* that downstream funders and investors require before committing to translational investment. Completing a training curriculum and producing an investor-grade FDA classification analysis or a validated user-requirements document are fundamentally different outputs, and it is the latter that gates access to major translational funding.

A meaningful advance in the field occurred with the development and deployment of the **G**uidance **a**nd **I**mpact **T**racking **S**ystem (GAITS), a web-based, maturity-model-driven commercialization framework originally developed to support the NIH Point-of-Care Technology Research Network (POCTRN) and subsequently scaled during the Rapid Acceleration of Diagnostics (RADx) Tech initiative, the NIH-funded program that brought the first FDA-authorized at-home COVID-19 test to market in under one year (13, 14). GAITS provides a comprehensive, validated taxonomy of commercialization maturity indicators organized across four translational domains—Technology, Regulatory, Market/Business, and Clinical—and links each indicator to recommended evidence requirements, associated risks, and actionable next steps as shown in Figure 1 (15).

**Figure 1:**
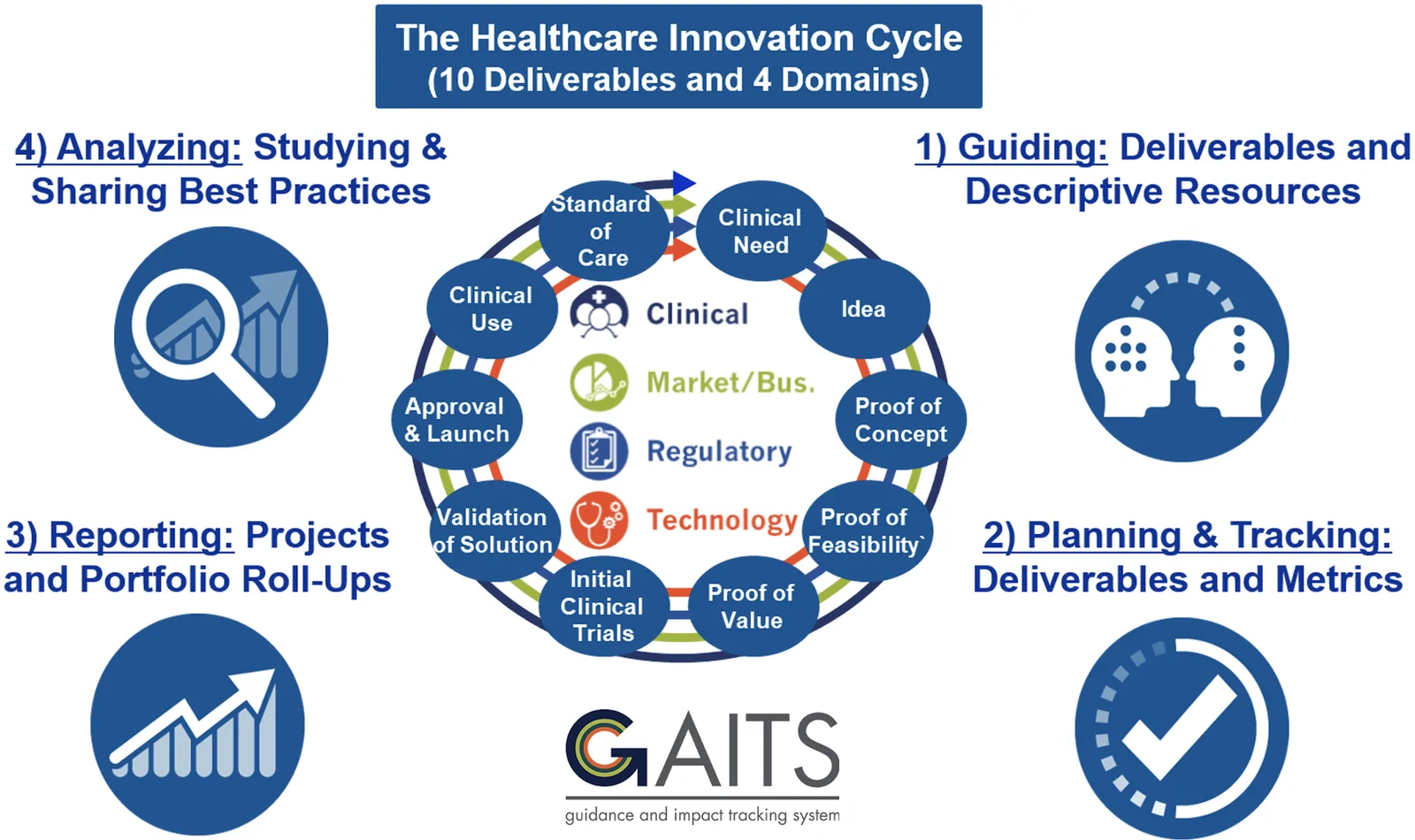
GAITS innovation cycle diagram.

Critically, GAITS functions both as a team-level guidance instrument and as a program-level portfolio management tool, enabling mentors and program administrators to track progress, identify bottlenecks, and allocate expert resources systematically across large, diverse project portfolios. Experience in RADx Tech shows that GAITS can be implemented across a large national portfolio under time-constrained conditions, providing operational precedent for its use in other NIH translational settings while not, by itself, establishing effectiveness or generalizability (14).

Building directly on this validated infrastructure, the NIH Blueprint MedTech Program was established to accelerate the development of transformative neurotechnologies from early proof-of-concept to first-in-human readiness, operating through two dedicated incubator hubs: the Center for Innovative NeuroTech Advancement (CINTA) at CIMIT and Spaulding Rehabilitation Hospital, and NeuroTech Harbor (*N*TH) at Johns Hopkins University and Howard University (16). Central to the Blueprint MedTech Program's architecture is the Seedling Award, a six-month, mentor-centered, GAITS-guided acceleration program—administered at Howard University—functioning as the gateway mechanism preceding the program's major investment award for most Blueprint MedTech applicants. The Seedling Program was designed explicitly for early-stage teams who demonstrate scientific or technical merit but exhibit significant uncertainty in one or more commercialization domains, precisely the profile of innovators underserved by existing training-based programs.

The defining innovation of the Seedling Program is its GAITS-Driven Gap-to-Deliverable (G2G) conversion framework: a structured process in which multidisciplinary expert reviewers evaluate submitted proposals, map identified commercialization weaknesses to specific GAITS maturity indicators across the four domains and translate these weaknesses into discrete Work Packages—time-bounded, evidence-generating bundles of deliverables achievable within the six-month program period. Work Packages are not training modules or reading assignments; they are actionable execution targets that produce the documentation, regulatory classification analyses, stakeholder and user requirements reports, competitive landscape assessments, reimbursement pathway summaries, risk files, and iterative prototype documentation, that downstream due-diligence processes require. This evidence-production orientation distinguishes Seedling from all prior HealthTech Innovation Commercialization Programs and represents a fundamental reconceptualization of how early-stage commercialization support should be delivered: not as knowledge transfer, but as structured, mentored, evidence generation aligned to established maturity criteria.

Blueprint MedTech completed two Seedling cohorts (Cohort 1: *N* = 17 teams; Cohort 2: *N* = 22 teams) and launched a third cohort of 19 teams in late 2025. In the completed cohorts, 13/17 teams (76.5%) and 18/22 teams (81.8%), respectively, advanced to Blueprint MedTech Deep Dive evaluation after Seedling. These proportions are reported as internal program progression metrics rather than as estimates of population-level neurotechnology translation success.

Although structured commercialization programs are increasingly used to support biomedical translation, few reports describe how reviewer-identified commercialization gaps are converted into time-bounded, evidence-generating deliverables within a neurotechnology-specific accelerator. The present manuscript describes the Seedling Program's design rationale, eligibility and selection criteria, GAITS-based gap assessment, Gap-to-Deliverable conversion process, mentorship and expert support architecture, and early program outcomes. We characterize cohort composition, gap burden, organizational and geographic distribution, neurological indications, technology categories, and progression to the program's Deep Dive stage. The evaluation is descriptive and is intended to provide an operational model and an internal benchmark for future controlled and longitudinal evaluation.

## 2 Materials and methods

### 2.1 Study design and program overview

This study is a descriptive program evaluation of the architecture, implementation, and early outcomes of the Blueprint MedTech Seedling Program across its first two completed cohorts (Cohort 1: *N* = 17 teams; Cohort 2: *N* = 22 teams), with selected descriptive characteristics also reported for Cohort 3. Quantitative analyses use administrative program records to summarize cohort characteristics, commercialization gap distributions, and Deep Dive progression. Process documentation and representative Work Package examples are used to describe program implementation; no formal thematic analysis or causal effect estimation was performed. The objective is to characterize the model and observed program outcomes, not to estimate a treatment effect (17, 18).

The Seedling Award functions as the gateway mechanism to the Blueprint MedTech ecosystem, designed to support early-stage neurotechnology teams that demonstrate scientific or technical merit but exhibit significant uncertainty in one or more commercialization domains. Each team receives $50,000 in program funds to support gap-closing activities, including consultant engagements, prototype development, stakeholder research, and regulatory analysis. Seedling is structured as a precursor to the program's major investment mechanism, the Blueprint MedTech Optimizer Award, which provides substantially larger resources for teams that have achieved demonstrable commercialization readiness.

### 2.2 Eligibility criteria and cohort selection

Eligibility criteria for Seedling participation were defined before each cohort. Selection required scientific or technical merit in a neurotechnology area relevant to Blueprint MedTech and at least one substantive commercialization weakness judged to be addressable within the six-month program. Eligible weaknesses included regulatory pathway uncertainty, team composition or commercialization expertise, market/business strategy, use-case and stakeholder alignment, technical or clinical proof-of-concept documentation, and project-management readiness. Thus, Seedling participants constitute a selected subgroup of applicants who had already passed scientific/technical and mission-fit screening and were judged to have remediable gaps; they should not be interpreted as a representative sample of all early-stage neurotechnology teams.

Proposals were excluded from Seedling eligibility if they lacked neurological or mental-health relevance to Blueprint MedTech's mission; did not demonstrate a functional prototype or credible initial technical feasibility; required more than six months of foundational scientific or engineering development before commercialization activities could meaningfully begin; or were sufficiently mature to proceed directly to in-depth due diligence evaluation without a structured readiness-building phase. These exclusion criteria ensured that Seedling resources were allocated to teams positioned to make substantive commercialization progress within the program's six-month window—neither too early to engage with GAITS-level deliverables nor too advanced to benefit from them.

Submitted applications underwent evaluation by a multidisciplinary review panel composed of clinical specialists, biomedical engineers, regulatory affairs professionals, product development strategists, and commercialization experts. Reviewer panels were assembled to ensure domain coverage across all four GAITS dimensions for each application. For every eligible team, the panel generated a structured feedback summary identifying specific commercialization uncertainties, risks, and underdeveloped domains—a document that formed the direct input to the subsequent gap-identification process. Cohort composition across both cohorts and the emerging third cohort is summarized in the Results section.

### 2.3 The GAITS framework: commercialization maturity across four domains

The Seedling Program uses GAITS, a web-based commercialization maturity framework that has been deployed across NIH-supported translational programs including POCTRN and RADx Tech. GAITS organizes commercialization readiness across four interdependent domains and ten maturity levels, linking maturity indicators to evidence requirements and recommended next steps (Figure 1).

Technology encompasses design controls, engineering feasibility, component integration, risk identification, and manufacturability planning. Maturity indicators in this domain address whether the device is engineered to a level where clinical and regulatory activities can proceed, and whether risk mitigation strategies are in place.

Regulatory encompasses FDA product classification, predicate device analysis, regulatory pathway selection [510(k), *de novo*, PMA, or exempt], definition of preclinical evidence requirements appropriate to the anticipated submission type, and strategies for early engagement with FDA's Center for Devices and Radiological Health (CDRH). Maturity indicators in this domain address whether a team has defined its regulatory strategy with sufficient specificity to estimate development cost, timeline, and risk, and, critically, whether that strategy is correctly matched to the device's intended use, technological characteristics, and risk classification rather than assumed by analogy to superficially similar products.

Market/Business encompasses value proposition definition, competitive landscape analysis, market sizing, reimbursement strategy, business model development, and stakeholder alignment across payers, health systems, and potential industry partners. Maturity indicators address whether teams understand the economic and market context sufficient to attract investment.

Clinical encompasses unmet clinical need definition, workflow integration analysis, user requirements elicitation, clinical evidence planning, and identification of key clinical champions and adoption stakeholders. Maturity indicators address whether teams have validated their clinical use case and understand the adoption pathway within real-world care settings.

Each GAITS indicator is linked to specific evidence requirements—concrete documentation, analyses, or outputs that demonstrate achievement of that maturity level—as well as associated risks if the indicator remains unaddressed and recommended actions for advancing to the next level. This structure transforms GAITS from an assessment checklist into a prescriptive, evidence-oriented guidance system (15). Critically, GAITS functions both at the team level, providing guidance to individual innovators and mentors, and at the portfolio level, enabling program administrators to track progress across cohorts, identify thematic bottlenecks, and allocate expert resources strategically.

### 2.4 GAITS-based commercialization gap identification

Following multidisciplinary review, Seedling program staff mapped reviewer-identified commercialization weaknesses to corresponding GAITS domains and maturity indicators. The unit of analysis for the cohort-level gap summaries was the team: a team was counted as having a gap category when at least one reviewer-identified weakness in that category was documented in the structured review record. Because a team could have gaps in more than one category, category percentages are not mutually exclusive and may sum to more than 100%.

Gap classification used a standardized four-domain GAITS taxonomy and the structured review summaries generated during application review. Program staff translated the documented weaknesses into team-specific gap lists that were then used to scope Work Packages. This was an operational program process rather than a blinded research coding exercise; inter-rater reliability was not prospectively measured. Future evaluations will use standardized scoring rules and duplicate coding to quantify reproducibility.

Gaps were documented using standardized GAITS terminology and translated to the GAITS platform, where they were accessible to the assigned mentor, program staff, and the team. This transparency ensured shared understanding of the program's objectives from the outset and allowed mentors to align their expertise and consultations directly with identified deficiencies rather than providing generalized commercialization guidance. Evidence documents were then uploaded for each Work Package to demonstrate progress. This created a repository that was easily accessible by team members, mentors, experts, and program staff.

### 2.5 Conversion of gaps to work packages: the core methodological innovation

The conversion of GAITS-derived gaps into structured Work Packages is the central operational feature of the Seedling Program (Figure 2). A Work Package is a time-bounded, evidence-generating bundle of approximately 2-6 related deliverables designed to address a specified gap by producing documentation, analyses, test results, or other artifacts aligned with the relevant GAITS maturity indicator (19, 20). Work Packages are therefore operational deliverable plans rather than educational modules. Representative deliverables are summarized in Table 1.

**Figure 2:**
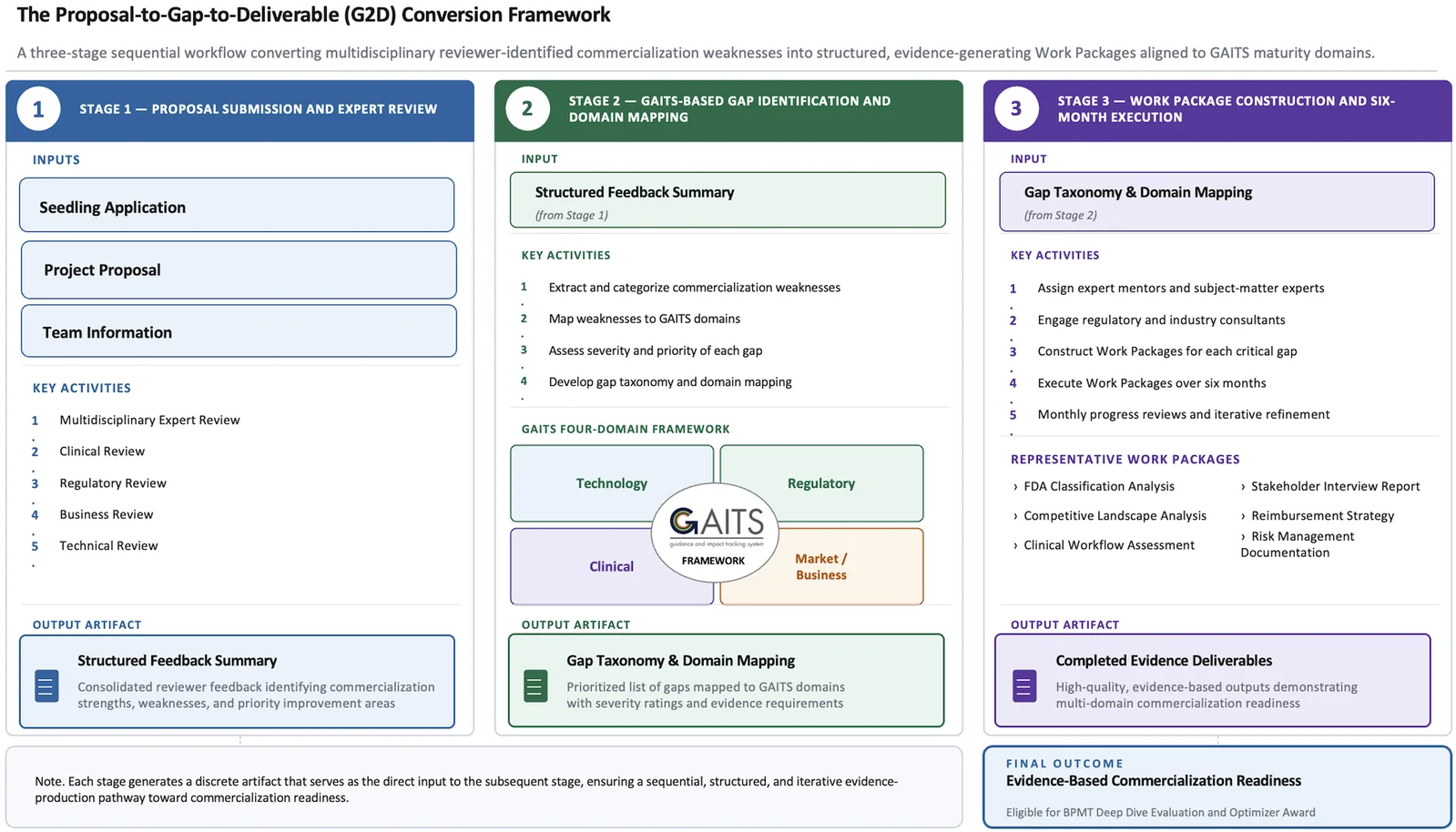
Gap to work package conversion in GAITS.

**Table 1:** Representative GAITS-aligned commercialization gaps and seedling work package deliverables.

GAITS domain	Representative gap	Representative Seedling deliverable	Evidence artifact/decision use
Technology	Engineering feasibility, risk, design controls, manufacturability	Risk analysis; design-control framework; bench-test plan/results; prototype iteration record	Documents technical risk reduction and readiness for later verification/validation
Regulatory	Uncertain classification, predicate, submission pathway, evidence requirements	FDA classification/predicate analysis; pre-submission plan; regulatory evidence map	Defines likely regulatory pathway and development evidence requirements
Market/Business	Unclear value proposition, market size, reimbursement, competitive position	Competitive landscape; market sizing; reimbursement/coding analysis; business-model/value proposition	Tests commercial assumptions and identifies adoption/payment constraints
Clinical	Unvalidated use case, workflow, stakeholder requirements, clinical evidence plan	Stakeholder interview synthesis; user requirements; workflow map; clinical evidence plan	Defines intended use context, user needs, adoption barriers, and evidence strategy

Work Packages assigned across Seedling cohorts have included, depending on the gap profile of each team: FDA product classification analyses and predicate device evaluations; structured stakeholder interview protocols and synthesized findings from interviews with target clinicians, patients, and payers; competitive landscape analyses with market sizing and positioning recommendations; reimbursement strategy formulations incorporating CPT, DRG, and HCPCS coding analysis; human factors and user requirement specifications aligned with FDA guidance on human factors engineering; risk analyses and preliminary design control documentation structured to ISO 14971 standards; clinical workflow mapping and care pathway analyses identifying integration points and adoption barriers; and early value proposition frameworks with economic modeling relevant to payer decision-making.

Each Work Package was constructed through a collaborative process involving the team, the assigned primary mentor, and program staff. Deliverables were scoped to be both achievable within the six-month program window and directly aligned with the GAITS maturity level the team was targeting. Work Packages were formally entered into the GAITS platform at program initiation and updated as deliverables were completed, providing a real-time progress record accessible to all program stakeholders. The $50,000 Seedling budget could be applied to support Work Package execution, including engagement of external consultants, procurement of market research resources, prototype development activities, and team training relevant to identified gaps.

### 2.6 Mentorship architecture and expert support infrastructure

Structured mentorship and expert consultation are integral components of the Seedling Program and are explicitly designed to support Work Package execution rather than to provide generalized commercialization education. Each team is assigned a Primary Mentor selected for domain-relevant expertise, which may be technical, clinical, regulatory, or business-focused, depending on the team's predominant gap profile. Primary mentors are engaged at program initiation and commit to a minimum of two structured engagements per month with their assigned team throughout the six-month program. These meetings are oriented around Work Package progress, deliverable quality review, and strategic guidance on gap-closing activities.

In addition to primary mentorship, each team receives a dedicated Regulatory Consultation of 90 min with a specialist in FDA medical device regulatory affairs, scheduled based on the team's regulatory gap profile and Work Package timeline. For teams with complex or ambiguous regulatory pathways, including novel neurotechnologies seeking *de novo* classification or combination product designation—this consultation is often among the highest-value program components, enabling precise definition of the regulatory strategy that informs all subsequent development and clinical planning.

Teams also access a network of Subject-Matter Experts whose engagements are scheduled on a needs-driven basis in alignment with specific Work Package requirements. Subject matter domains include human factors engineering, reimbursement strategy, market research methodology, clinical trial design, intellectual property, and quality management systems. Monthly Neurotech Education Webinars covering translational topics relevant to the cohort's collective gap profile supplement individual mentor and Subject Matter Expert engagements, providing shared learning opportunities across the cohort. Throughout the program, Blueprint MedTech Seedling staff at Howard University are available for GAITS interpretation, deliverable scoping, and program navigation support.

This layered support architecture, combining longitudinal primary mentorship, targeted regulatory consultation, on-demand Subject Matter Expert access, and structured educational programming, ensures that teams have the intellectual and advisory resources required to execute their Work Packages to GAITS maturity standards within the six-month timeline.

### 2.7 Data sources and primary variables

Data for this study were derived from multiple complementary sources. Structural program documentation, including eligibility criteria, program flowcharts, and the GAITS-derived gap taxonomy, provided the foundation for describing program design. Cohort-level data on gap distributions, organizational characteristics, demographic profiles, targeted neurological indications, and technological categories were derived from program-generated classification documents. Downstream progression outcomes for Cohorts 1 (*N* = 17) and 2 (*N* = 22) were derived from program decision records documenting subsequent in-depth due-diligence evaluation outcomes for each team following Seedling completion.

### 2.8 Outcome definitions

The primary outcome was progression to Blueprint MedTech in-depth due-diligence evaluation (‘Deep Dive’) after Seedling completion, defined as a formal invitation to undergo review for potential Optimizer Award funding. Deep Dive progression is a binary, proximal program endpoint. The Deep Dive reviewers are distinct from the Seedling mentors and day-to-day program staff; however, the Deep Dive process occurs within the same Blueprint MedTech ecosystem and evaluates readiness using criteria that are intentionally aligned with the GAITS-informed work completed during Seedling. Accordingly, this endpoint is best interpreted as an internal program readiness benchmark, not as independent external validation of commercial or clinical success.

Secondary outcomes were the distribution of commercialization gap categories, organizational and demographic composition, geographic distribution, targeted neurological indications, and technology categories. Work Package records were used descriptively to provide examples of deliverables completed across GAITS domains. Because standardized pre/post GAITS scores were not available for Cohorts 1 and 2, the present study does not quantify within-team changes in maturity.

### 2.9 Data analysis

Counts, proportions, and distributions were generated from program records. Progression rates are reported with exact Clopper-Pearson 95% confidence intervals. The Cohort 1 and Cohort 2 progression proportions were compared descriptively and with a two-sided Fisher exact test as an exploratory analysis appropriate for small cell counts. No adjustment for covariates or multiplicity was performed, and no causal interpretation was assigned to between-cohort differences. Gap, demographic, organizational, indication, and technology summaries are presented as counts and proportions where available.

Work Package descriptions and program narratives are presented only as representative operational examples. They were not subjected to formal thematic analysis and are not treated as independent evidence of program effectiveness. Participant-reported outcomes, satisfaction, and longer-term impact are outside the scope of this analysis.

### 2.10 Ethical and administrative considerations

This work used de-identified administrative records generated during routine operation of a federally funded translational research program. No protected health information or individually identifiable private information was analyzed. The activity was treated as program evaluation rather than human-subjects research under 45 CFR 46. The Institutional Review Board determined that this program evaluation was exempt/non-human-subjects research. Several authors participated in the design, administration, mentorship, or evaluation of the Seedling Program. Quantitative outcomes were therefore anchored to formal program decision records, and qualitative examples were used descriptively; nevertheless, the internal-evaluator role remains a potential source of interpretive bias and is acknowledged in the Limitations.

## 3 Results

### 3.1 The blueprint MedTech funding pipeline and seedling's role as gateway accelerator

The Blueprint MedTech funding pipeline is structured as a sequential, multi-stage evaluation process in which the Seedling Program serves as a structured readiness-building intervention embedded within the broader review pathway (Figure 3). The pipeline begins with a solicitation inviting submissions from early-stage neurotechnology teams. Submitted applications first undergo a preproposal stage, followed by a mission-fit screen that evaluates neurological and neurotechnology relevance. Teams passing mission-fit screening are invited to submit a full proposal, which is then reviewed by the Viability Panel. For teams that demonstrate scientific merit but exhibit substantive commercialization uncertainties across regulatory, market, clinical, or technical domains, the Viability Panel directs teams into the Seedling Program. As depicted in Figure 3, upon completing the Seedling program, each team is evaluated at a decision point with three possible outcomes:

**Figure 3:**
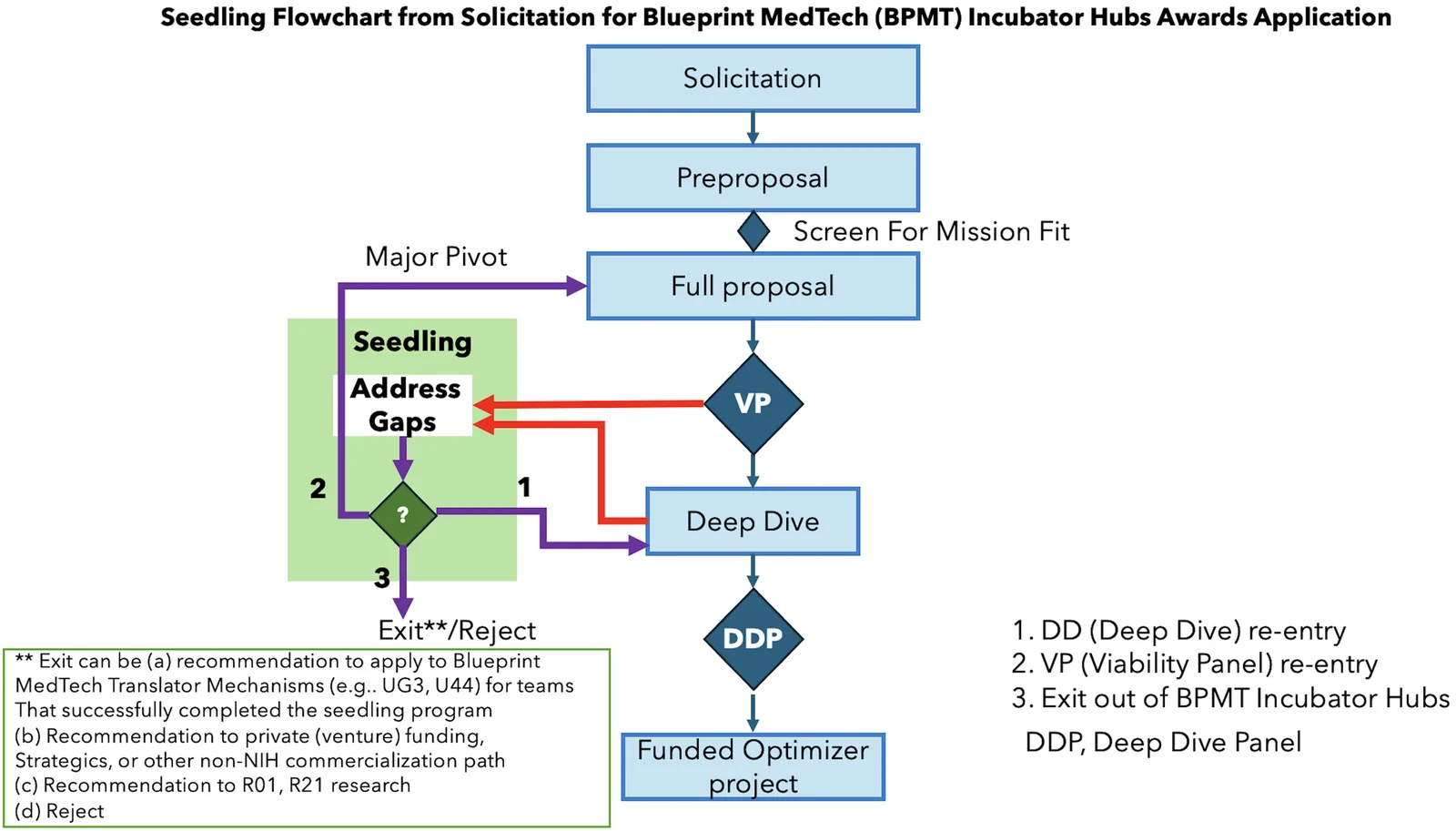
The BPMT funding pipeline.

Deep Dive: Teams that demonstrate sufficient commercialization maturity through their Work Package deliverables advance directly into Deep Dive evaluation, bypassing the need for another full Viability Panel review. This is the primary intended outcome of Seedling participation and the pathway taken by 31 of 39 teams (79.5%) across Cohorts 1 and 2.

Viability Panel: Teams that have made meaningful progress during Seedling but have not yet achieved the evidence threshold required for Direct Deep Dive advancement are directed back to the Viability Panel for re-evaluation. Viability Panel acknowledges that some teams require additional deliberation at the panel level before the decision to commit Deep Dive resources is made, rather than an outright rejection of further participation; and

Exit/Reject: Teams that do not demonstrate sufficient progress or commercial viability to justify continued investment in the Blueprint MedTech pipeline exit the Seedling phase. Importantly, exit is not equivalent to simple rejection: teams exiting the Seedling program are directed to one of four constructive forward pathways—(a) application to Blueprint MedTech Translator Mechanisms such as UG3 or U44 awards for teams that successfully completed Seedling but are not yet Optimizer Award-ready; (b) recommendation to pursue private or venture funding, strategic industry partnerships, or other non-NIH commercialization paths; (c) recommendation to pursue additional basic research through R01 or R21 grant mechanisms before re-engaging the translational pipeline; or (d) formal rejection from the Blueprint MedTech ecosystem. This multi-pathway exit architecture reflects the program's commitment to supporting the broader neurotechnology innovation ecosystem beyond its own funding funnel.

Teams invited to Deep Dive are evaluated by a reviewer panel that is distinct from the Seedling mentorship team and the Viability Panel. The panel remains part of the Blueprint MedTech program and therefore should be viewed as organizationally separate rather than fully external to the program. It makes recommendations regarding progression toward the Funded Optimizer Project.

### 3.2 Cohort composition: scale, scope, and growth across three cycles

Across the three Seedling cohorts initiated to date, the program supported 58 teams: 17 in Cohort 1 (completed 2024), 22 in Cohort 2 (completed 2025), and 19 in Cohort 3 (launched October 2025). The present outcome analysis is limited to the two completed cohorts (*N* = 39). Cohort 3 is included only in descriptive summaries where complete data were available; no inference is made regarding reasons for cohort-size differences.

### 3.3 Seedling gap distributions: systemic and multi-domain deficiencies at program entry

Structured Viability Panel review identified commercialization weaknesses across multiple domains at program entry (Figure 4). These gaps were documented by multidisciplinary reviewers rather than self-reported by applicant teams. Because the reviews occurred within the Blueprint MedTech selection process, the resulting gap profile is an internal structured assessment of the selected Seedling population rather than an externally validated prevalence estimate.

**Figure 4:**
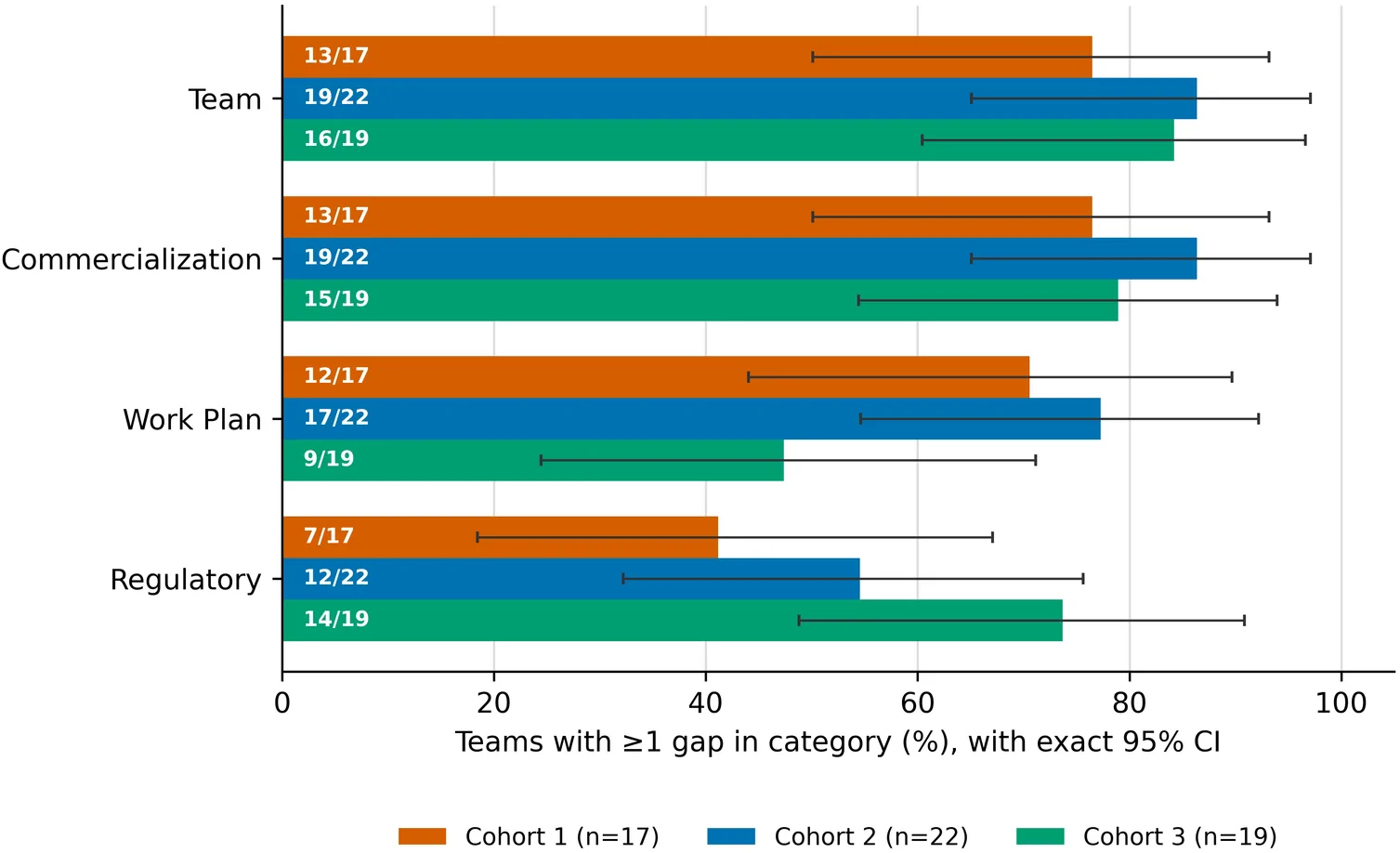
Gap distributions across cohorts.

Across all 58 teams in Cohorts 1-3, team-composition gaps were identified in 48 teams (82.8%): 13/17 (76.5%) in Cohort 1, 19/22 (86.4%) in Cohort 2, and 16/19 (84.2%) in Cohort 3. Common examples included absence of dedicated regulatory expertise, insufficient clinical-domain expertise for the target indication, lack of a business/commercialization lead, or an imbalance between scientific/engineering expertise and translational capabilities.

Commercialization gaps were identified in 47/58 teams (81.0%): 13/17 (76.5%) in Cohort 1, 19/22 (86.4%) in Cohort 2, and 15/19 (78.9%) in Cohort 3. Examples included incomplete competitive-landscape analysis, undefined reimbursement strategy, insufficient market sizing or segmentation, and an underdeveloped business model.

Work Plan gaps were identified in 38/58 teams (65.5%): 12/17 (70.6%) in Cohort 1, 17/22 (77.3%) in Cohort 2, and 9/19 (47.4%) in Cohort 3. These gaps included underdeveloped milestone structures, unclear deliverables, insufficient description of technical feasibility within the proposed timeline, and inadequately scoped resource allocation. The cohort-specific percentages are presented descriptively and are not interpreted as evidence of a temporal trend.

Regulatory gaps were identified in 33/58 teams (56.9%): 7/17 (41.2%) in Cohort 1, 12/22 (54.5%) in Cohort 2, and 14/19 (73.7%) in Cohort 3. These descriptive differences may reflect cohort composition, review practices, or other unmeasured factors; the present data do not establish a longitudinal trend.

Across Cohorts 1 and 2, the two cohorts for which individual-level gap data are available, 88% of Cohort 1 teams (15/17) and 100% of Cohort 2 teams (22/22) exhibited gaps in two or more domains simultaneously.

### 3.4 Geographic distribution: national reach across diverse innovation ecosystems

Seedling teams were geographically distributed across multiple U.S. regions (Figure 5), including the Northeast, West Coast, Midwest, and Southeast. The distribution demonstrates that the program enrolled teams from multiple innovation ecosystems; without data on the geographic distribution of all applicants or an external comparison population, it cannot establish comparative accessibility or equity.

**Figure 5:**
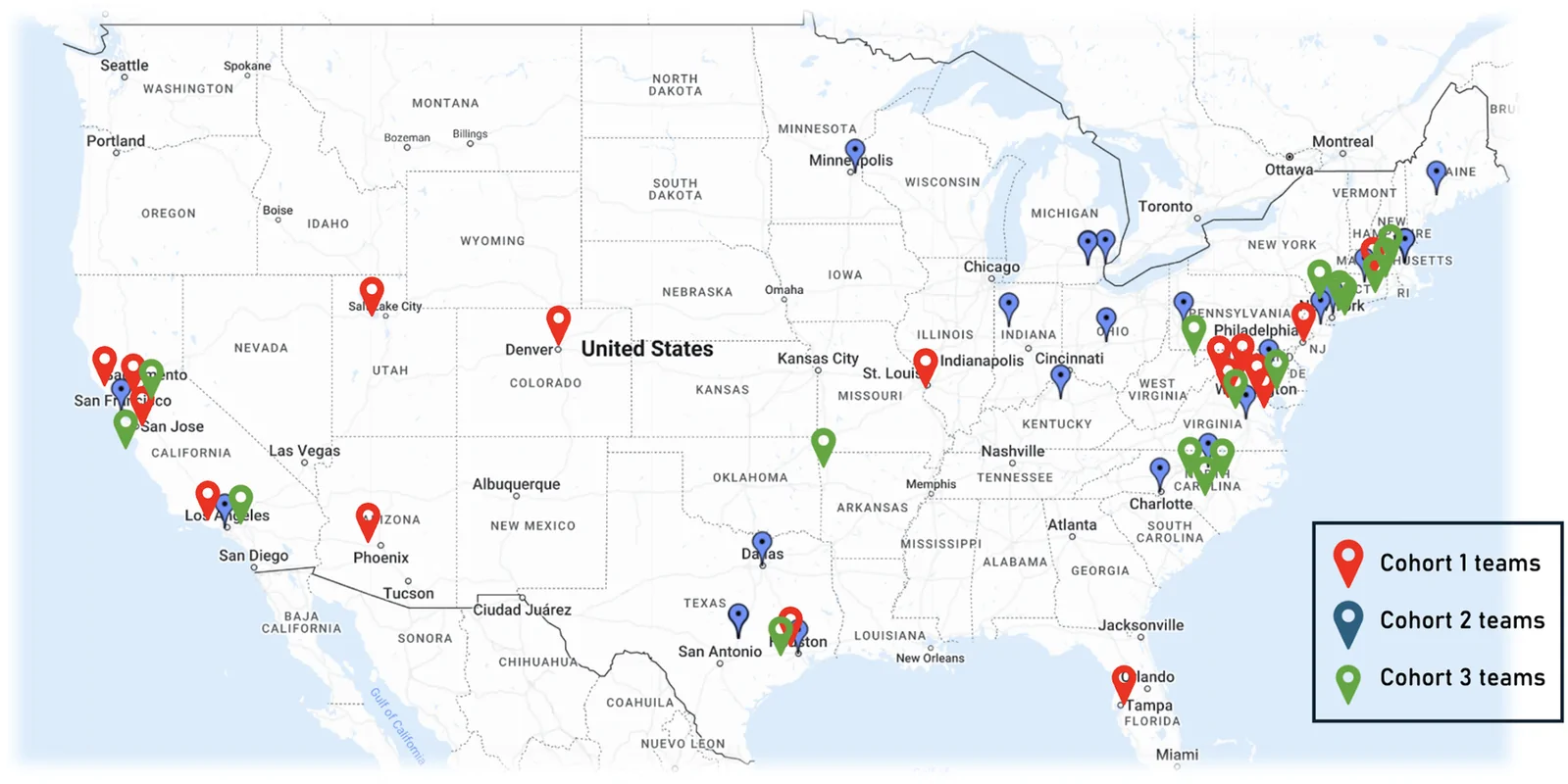
Geographic distribution.

The multi-region distribution is operationally relevant because Seedling mentorship and expert consultation can be delivered remotely without geographic co-location. Whether this model improves access relative to other commercialization pathways is an important question for future comparative evaluation.

### 3.5 Demographic characteristics of cohort 2

Comprehensive demographic representation data were available for Cohort 2 (*N* = 22) and are therefore reported descriptively for that cohort only (Figure 6). Data are summarized separately for leadership and broader team membership across the categories captured in program records. No external or applicant-pool comparator was available.

**Figure 6:**
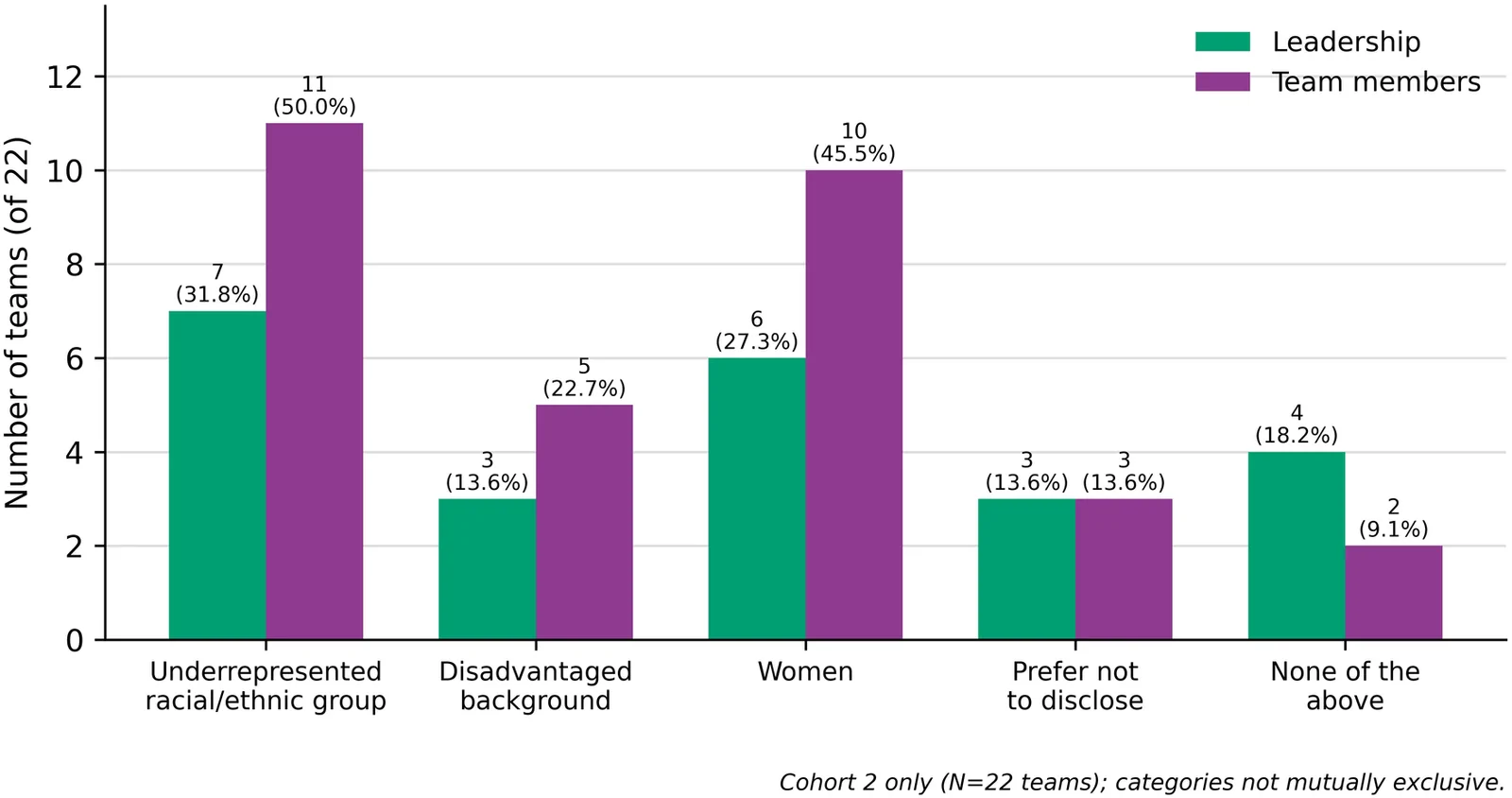
Demographic characteristics of cohort 2.

Among team Leadership, 7 of 22 teams (31.8%) were led by individuals from underrepresented racial or ethnic groups, 6 of 22 (27.3%) were led by women, 3 of 22 (13.6%) reported a disadvantaged background, and 3 of 22 (13.6%) preferred not to disclose their demographic status. Four teams (18.2%) reported none of the represented categories in leadership.

Among Team Members, 11 teams (50.0%) included members from underrepresented racial or ethnic groups, 10 (45.5%) included women, 5 (22.7%) included individuals from disadvantaged backgrounds, and 3 (13.6%) preferred not to disclose. Two teams (9.1%) reported none of the represented categories among their membership.

These data describe the composition of Cohort 2 but do not establish that Seedling increased representation, improved equity, or was more accessible than alternative pathways. Those questions require comparison with the applicant pool and other relevant translational programs.

### 3.6 Organizational composition of seedling cohorts

Across Cohorts 1-3, teams were categorized in program records as academic institutions (26/58; 44.8%), small businesses with 50 or fewer employees (16/58; 27.6%), or start-ups less than one year old (16/58; 27.6%) (Figure 7). These categories describe the organizational composition of the portfolio; the present analysis did not test whether gap profiles or progression differed by organization type.

**Figure 7:**
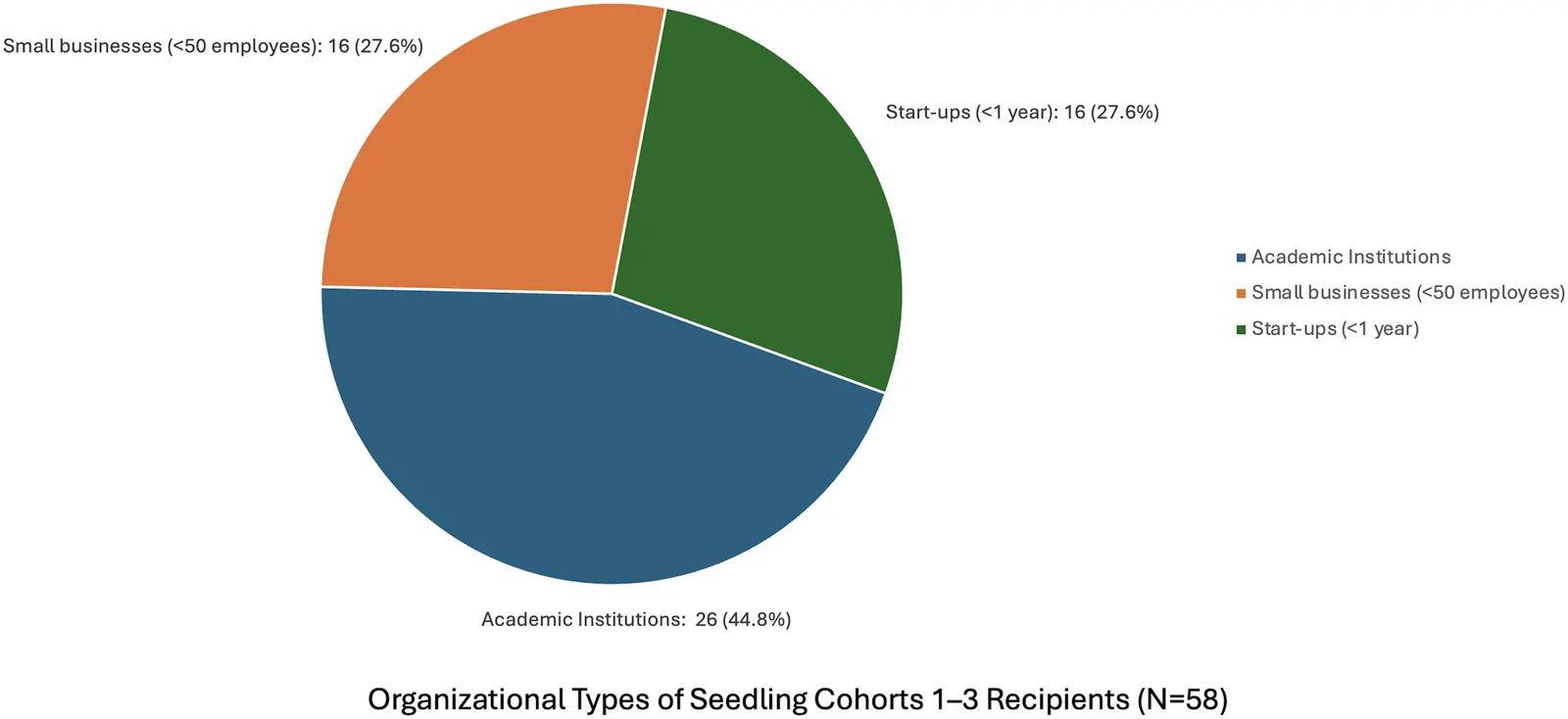
Organizational landscape here.

### 3.7 Neurological and psychiatric indications: broad clinical relevance across the nervous system

Targeted neurological and psychiatric conditions spanned a broad range of indications (Figure 8), including pain, epilepsy, stroke, substance use disorder, spinal cord injury, Parkinson's disease, traumatic brain injury, peripheral nerve injury, cerebral palsy, depression, neurodegenerative conditions, and others. This distribution demonstrates breadth of indication coverage within the Seedling portfolio; it does not by itself establish indication-independent effectiveness of the program.

**Figure 8:**
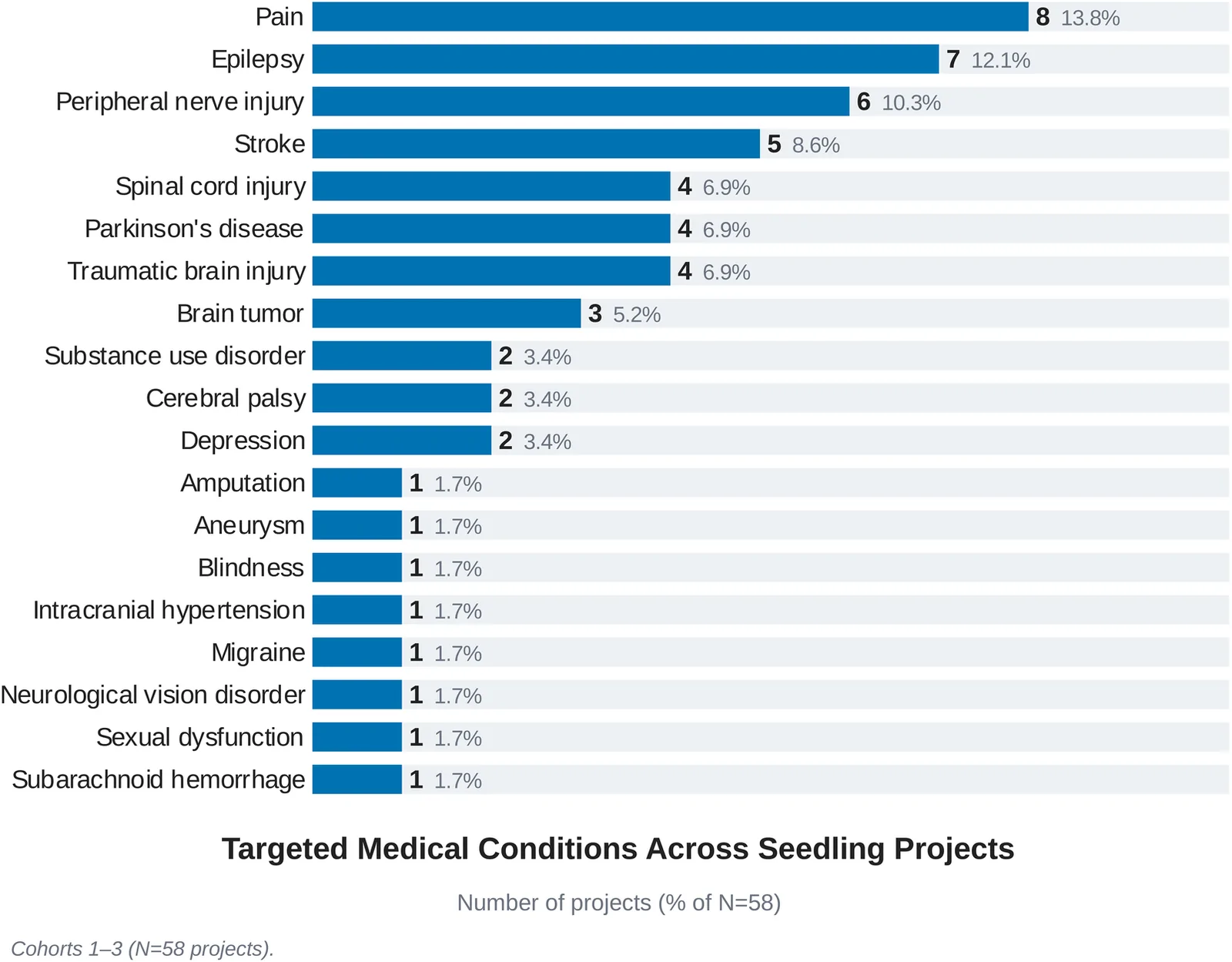
Targeted medical conditions across seedling projects.

### 3.8 Technological categories: diverse device and digital health platforms

Technologies represented in the Seedling portfolio included neuromodulation, ultrasound and imaging, near-infrared spectroscopy, wearables, functional electrical stimulation, biofeedback, decision-support tools, artificial intelligence, prosthetics, catheter-based neurophysiology, drug-delivery systems, and other device categories (Figure 9). The portfolio therefore included hardware, software-enabled, and hybrid technologies with heterogeneous regulatory and development pathways.

**Figure 9:**
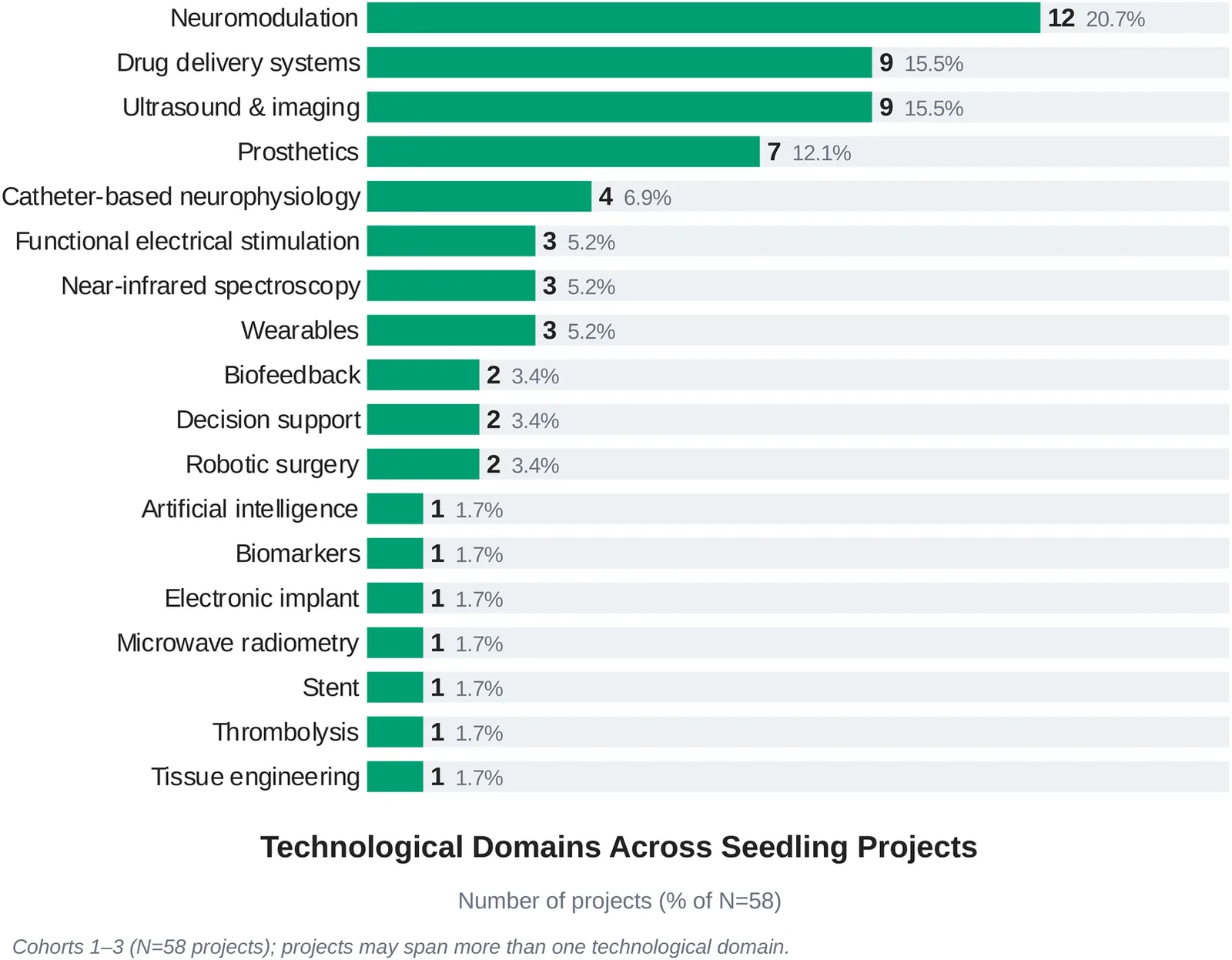
Technological domains.

This technological breadth suggests that the GAITS taxonomy can be operationalized across diverse device types, but the current single-program evaluation does not establish that the G2G model performs equivalently across technology classes. Future analyses with standardized pre/post scoring and larger samples should test that question directly.

### 3.9 Primary outcome: progression to blueprint MedTech deep dive evaluation

The primary outcome measure—progression to Blueprint MedTech Deep Dive panel evaluation following completion of Seedling Work Packages—is summarized for both completed cohorts in Table 2. Cohort-specific estimates are presented with exact 95% confidence intervals and are interpreted descriptively.

**Table 2:** Seedling program progression outcomes across cohorts 1 and 2.

Cohort	N Teams	Advanced to Deep Dive	Did Not Advance	Progression Rate	Exact 95% CI
Cohort 1	17	13	4	76.5%	50.1%–93.2%
Cohort 2	22	18	4	81.8%	59.7%–94.8%
Combined	39	31	8	79.5%	63.5%–90.7%

Blueprint MedTech Seedling Program progression outcomes across Cohorts 1 and 2. Progression is defined as advancement to the internal BPMT Deep Dive panel evaluation following completion of GAITS-aligned Work Packages. Exact 95% confidence intervals are Clopper-Pearson intervals. The Cohort 1 versus Cohort 2 progression proportions did not differ statistically (two-sided Fisher exact *p* = 0.709).

In Cohort 1 (*N* = 17), 13 teams advanced to Deep Dive (76.5%; exact 95% CI, 50.1%–93.2%) and 4 did not. In Cohort 2 (*N* = 22), 18 teams advanced (81.8%; 95% CI, 59.7%–94.8%) and 4 did not. Across both cohorts, 31/39 teams advanced (79.5%; 95% CI, 63.5%–90.7%) (Table 2). These values are internal program progression rates among pre-screened Seedling participants and are not directly comparable with population-level medical-device development success rates.

The 5.3-percentage-point difference between Cohort 1 and Cohort 2 was not statistically significant (two-sided Fisher exact *p* = 0.709), and the confidence intervals overlap substantially. The observed difference is therefore compatible with no true between-cohort difference and is not interpreted as evidence of program improvement over time.

Deep Dive progression is a demanding internal gate, but it remains a proximal outcome within the same Blueprint MedTech ecosystem. The high observed progression proportion indicates that many selected teams met the program's next-stage readiness criteria after Seedling; it does not establish that Seedling caused the progression or predict regulatory approval, commercial launch, clinical adoption, or patient benefit.

### 3.10 GAITS-aligned deliverables completed across domains

Program records document completion of GAITS-aligned deliverables across all four domains. In the Regulatory domain, examples included FDA product-classification analyses, predicate-device assessments, pre-submission planning, and clarification of potential *de novo* or combination-product requirements. These examples indicate the types of artifacts generated during Seedling rather than a quantified pre/post change in regulatory maturity.

In the Market/Business domain, representative deliverables included competitive-landscape analyses, market-sizing work, reimbursement pathway analyses, and value-proposition documents. In the Clinical domain, examples included stakeholder interview summaries, user-requirements documents, workflow maps, and clinical evidence plans. In the Technology domain, examples included risk analyses, design-control documentation, and bench-testing plans or results where applicable.

Because Cohorts 1 and 2 did not have standardized, scored GAITS assessments at both entry and exit, these records should be interpreted as documentation of deliverable completion rather than quantitative evidence of a measured maturity gain. Prospective pre/post GAITS scoring is planned for subsequent cohorts.

## 4 Discussion

### 4.1 Principal findings

#### 4.1.1 High internal progression among selected seedling participants

This program evaluation describes a structured GAITS-driven Gap-to-Deliverable model and reports a high rate of progression to the next internal Blueprint MedTech review stage among selected Seedling participants. Across 39 teams in two completed cohorts, 31 (79.5%; 95% CI, 63.5%–90.7%) advanced to Deep Dive after Seedling. Because participants were pre-screened for scientific/technical merit and addressable commercialization gaps, there was no untreated comparator, and the endpoint was an internal program decision aligned with the intervention's deliverables, this proportion should be interpreted as a descriptive program benchmark rather than a causal estimate of effectiveness.

Three descriptive findings are particularly informative. First, multi-domain commercialization gaps were common at entry: team composition in 82.8%, commercialization strategy in 81.0%, work planning in 65.5%, and regulatory strategy in 56.9% of the 58 teams. Second, Seedling was implemented across geographically and organizationally diverse teams and across a broad range of indications and technologies. Third, the operational records demonstrate that reviewer-identified gaps can be translated into concrete, time-bounded evidence deliverables across GAITS domains. The data do not establish that the model caused improved readiness, nor do they support an inference of improvement from Cohort 1 to Cohort 2.

The primary contribution of the present report is therefore methodological and operational: it documents a reproducible approach for converting structured commercialization gaps into mentored, evidence-generating Work Packages and provides an internal benchmark against which future prospective and comparative evaluations can be designed.

### 4.2 The seedling model in the context of existing HealthTech commercialization programs

The Seedling Program shares elements with established HealthTech commercialization programs while placing greater emphasis on required evidence artifacts. I-Corps and I-Corps at NIH emphasize customer discovery and iterative business-model testing (11, 21), whereas C3i provides structured commercialization education spanning regulatory, reimbursement, and development topics. CRAASH combines customer discovery with GAITS-informed maturity assessment and expert facilitation. These programs differ in scope, duration, funding, participant selection, and required outputs, limiting direct outcome comparisons.

Seedling adds a specific operational requirement: selected teams are assigned GAITS-aligned Work Packages whose completion requires tangible evidence artifacts such as regulatory analyses, user-requirements documentation, stakeholder findings, market/reimbursement analyses, or technical risk documentation. This emphasis on deliverable completion is a distinction in program design; it should not be interpreted as evidence that Seedling is superior to other programs without direct comparative data.

The G2G framework can therefore be viewed as a structured remediation model in which weaknesses identified during review are translated into defined deliverables and revisited during mentorship. The alignment between Work Packages and downstream Blueprint criteria is intentional and operationally useful, but it also contributes to criterion contamination in the present outcome measure, as discussed below.

### 4.3 Why structured evidence production is particularly consequential in neurotechnology

Neurotechnology development often requires simultaneous attention to device engineering, regulatory classification, human factors, clinical workflow, evidence planning, reimbursement, and market adoption (22, 23). The Seedling gap profiles illustrate that selected teams frequently entered with uncertainty in several of these domains. The program's structured Work Packages were designed to make those uncertainties explicit and convert them into concrete tasks.

The potential value of identifying such issues early is practical rather than proven by the present data: an incorrect regulatory assumption, poorly specified clinical use case, or untested workflow assumption can require redesign or delay later development. The current study documents that Seedling systematically addressed these issues; future longitudinal comparisons are needed to determine whether doing so shortens timelines, reduces costs, or improves downstream success.

Accordingly, the present findings support feasibility of structured, front-loaded evidence generation across heterogeneous neurotechnology projects, but they do not quantify its causal effect on translational outcomes.

### 4.4 GAITS as program infrastructure

#### 4.4.1 Lessons from RADx tech and POCTRN

GAITS provides common terminology, maturity indicators, and portfolio-level tracking across Technology, Regulatory, Market/Business, and Clinical domains. It has been implemented in POCTRN and RADx Tech, where it supported consistent assessment and work-plan tracking across large, heterogeneous portfolios (14, 24, 25). These precedents demonstrate feasibility of implementation across large portfolios, but they do not establish the scalability, effectiveness, or generalizability of the Seedling implementation.

In Seedling, alignment between GAITS-defined deliverables and the criteria considered during Blueprint due diligence provides coherence between remediation and the next program gate. At the same time, because the primary outcome is assessed within the same institutional framework, the observed progression rate may partly reflect this designed criterion alignment. The endpoint therefore should not be treated as independent external validation.

GAITS also permits program-level aggregation of recurring gap categories and may support future hypothesis generation regarding which gap profiles are associated with downstream outcomes. Such predictive or comparative analyses will require larger samples, standardized pre/post scoring, prespecified definitions, and validation outside the program in which the model was developed.

### 4.5 Demographic composition and future equity evaluation

Cohort 2 included women, individuals from racial or ethnic groups categorized as underrepresented in program records, and individuals reporting disadvantaged backgrounds. These data demonstrate representation within the cohort but cannot establish that Seedling improved equity, democratized access, or achieved greater representation than the applicant pool or other commercialization programs because no comparator data were available.

Equity is therefore best framed as a testable program objective rather than an observed impact of the present evaluation (26). Future analyses should compare the demographic composition of applicants, selected Seedling teams, non-selected applicants, and relevant external programs; examine whether selection and progression rates differ across groups; and assess whether GAITS-based Work Packages produce comparable readiness changes when standardized pre/post measures are available.

Systematic demographic data collection across all future cohorts will be required before conclusions about program accessibility or equity can be made.

### 4.6 Potential transferability to other translational programs

The gap categories addressed by Seedling—regulatory uncertainty, market/business planning, team composition, clinical use-case definition, and milestone planning—are also relevant to other medical-device and digital-health programs. GAITS provides a domain-general structure that can be adapted to different technology classes, while Work Packages can be calibrated to the regulatory, clinical, and market requirements of a specific domain.

Experience with GAITS in POCTRN and RADx Tech suggests that the framework can be implemented across different translational contexts. The Seedling findings add a neurotechnology-specific example, but the present study does not demonstrate that the same progression rates or benefits would occur in other NIH institutes, technology domains, or accelerator structures. Replication and comparative evaluation are needed before broad generalizability is claimed.

Cost-effectiveness and financial leverage were not evaluated in the present analysis because follow-on funding data were incomplete and not collected with a design suitable for rigorous economic evaluation.

### 4.7 Limitations and considerations

Several limitations constrain interpretation. First, Deep Dive progression is a proximal, program-specific endpoint. Reviewers at Deep Dive are distinct from Seedling mentors, but they operate within the same Blueprint MedTech ecosystem and assess criteria that are intentionally aligned with GAITS-informed deliverables. This creates potential criterion contamination: Seedling is designed to prepare teams for the same readiness dimensions used at the next gate. The endpoint therefore reflects internal program readiness rather than independent evidence of regulatory, commercial, clinical, or patient-level success.

Second, there is no untreated or matched comparison group, and Seedling participation is highly selected. Teams had already been screened for mission fit and scientific/technical merit and were chosen because their commercialization gaps were judged addressable within six months. These selection factors may independently predict downstream progression. Consequently, the 79.5% rate applies only to this selected Seedling population and cannot be interpreted as a population-level translation rate or a causal program effect. We therefore removed the prior comparison with a 20%–30% historical benchmark, which was not based on a directly comparable population or outcome.

Third, standardized quantitative GAITS scores were not collected at both entry and exit for Cohorts 1 and 2, preventing direct measurement of within-team maturity change. Work Package completion records document outputs but do not provide a validated effect-size estimate of readiness gain.

Fourth, comprehensive demographic data were available only for Cohort 2, and no applicant-pool or external comparator was available. The demographic findings therefore describe cohort composition only and cannot support conclusions about equity impact.

Fifth, cohort sizes were modest (*N* = 17 and *N* = 22), yielding wide confidence intervals around progression estimates and limited power for subgroup or between-cohort comparisons. Sixth, several authors were directly involved in program design, administration, or evaluation, creating potential internal-evaluator bias despite use of formal administrative records for the primary outcome.

### 4.8 Future directions

Future evaluation should prioritize longitudinal external outcomes, including Optimizer funding decisions, follow-on non-Blueprint funding, first-in-human studies, regulatory submissions or clearances, clinical adoption, and other prespecified milestones. These outcomes will permit assessment of whether internal Deep Dive progression predicts meaningful downstream translation.

Prospective standardized GAITS scoring at program entry and exit should be implemented with explicit scoring rules, duplicate assessment of a subset of teams, and reporting of inter-rater agreement. These data would support domain-specific effect-size estimation and more reproducible evaluation of readiness changes.

Comparative analyses should examine appropriately matched Blueprint MedTech applicants who did not participate in Seedling, where feasible, or use other quasi-experimental designs that address selection. Replication in independent translational programs would test transferability. Predictive analytics or AI-assisted gap detection should be considered only after the underlying definitions, labels, and outcomes are standardized and externally validated (27).

## 5 Conclusion

The Blueprint MedTech Seedling Program operationalizes a GAITS-driven Gap-to-Deliverable framework that converts reviewer-identified commercialization weaknesses into time-bounded evidence artifacts. Among pre-screened teams in the first two completed cohorts, 79.5% advanced to the program's internal Deep Dive stage. This high internal progression rate is a useful program benchmark, but the selected single-arm design, criterion-aligned endpoint, and absence of standardized pre/post maturity scores preclude causal claims of effectiveness. Prospective scoring, external outcomes, and comparative designs are needed to determine whether and to what extent the G2G model improves downstream neurotechnology translation.

## Data Availability

The datasets presented in this article are not readily available because the datasets analyzed in this study are not publicly available because they contain proprietary program evaluation data and information that could identify participating teams. De-identified data may be made available by the corresponding author upon reasonable request, subject to institutional approval. Requests to access the datasets should be directed to Evaristus Nwulia, enwulia@howard.edu.
